# Mollusc genomes reveal variability in patterns of LTR-retrotransposons dynamics

**DOI:** 10.1186/s12864-018-5200-1

**Published:** 2018-11-15

**Authors:** Camille Thomas-Bulle, Mathieu Piednoël, Tifenn Donnart, Jonathan Filée, Didier Jollivet, Éric Bonnivard

**Affiliations:** 1 Sorbonne Université, Univ Antilles, CNRS, Institut de Biologie Paris Seine (IBPS), Laboratoire Evolution Paris Seine, F-75005 Paris, France; 2 Sorbonne Université, CNRS, UMR 7144 AD2M, Station Biologique de Roscoff, Place Georges Teissier CS90074, 29688 Roscoff, France; 3 Laboratoire Evolution, Génomes, Comportement, Ecologie; CNRS, IRD, Université Paris-Saclay, Gif-sur-Yvette, France

**Keywords:** LTR- retrotransposons, Gypsy, Copia, BEL/Pao, Molluscs, Comparative genomic

## Abstract

**Background:**

The three superfamilies of Long Terminal Repeat (LTR) retrotransposons are a widespread kind of transposable element and a major factor in eukaryotic genome evolution. In metazoans, recent studies suggested that Copia LTR-retrotransposons display specific dynamic compared to the more abundant and diverse Gypsy elements. Indeed, Copia elements show a relative scarcity and the prevalence of only a few clades in specific hosts. Thus, BEL/Pao seems to be the second most abundant superfamily. However, the generality of these assumptions remains to be assessed. Therefore, we carried out the first large-scale comparative genomic analysis of LTR-retrotransposons in molluscs. The aim of this study was to analyse the diversity, copy numbers, genomic proportions and distribution of LTR-retrotransposons in a large host phylum.

**Results:**

We compare nine genomes of molluscs and further added LTR-retrotransposons sequences detected in databases for 47 additional species. We identified 1709 families, which enabled us to define 31 clades. We show that clade richness was highly dependent on the considered superfamily. We found only three Copia clades, including GalEa and Hydra which appear to be widely distributed and highly dominant as they account for 96% of the characterised Copia elements. Among the seven BEL/Pao clades identified, Sparrow and Surcouf are characterised for the first time. We find no BEL or Pao elements, but the rare clades Dan and Flow are present in molluscs. Finally, we characterised 21 Gypsy clades, only five of which had been previously described, the C-clade being the most abundant one. Even if they are found in the same number of host species, Copia elements are clearly less abundant than BEL/Pao elements in copy number or genomic proportions, while Gypsy elements are always the most abundant ones whatever the parameter considered.

**Conclusions:**

Our analysis confirms the contrasting dynamics of Copia and Gypsy elements in metazoans and indicates that BEL/Pao represents the second most abundant superfamily, probably reflecting an intermediate dynamic. Altogether, the data obtained in several taxa highly suggest that these patterns can be generalised for most metazoans. Finally, we highlight the importance of using database information in complement of genome analyses when analyzing transposable element diversity.

**Electronic supplementary material:**

The online version of this article (10.1186/s12864-018-5200-1) contains supplementary material, which is available to authorized users.

## Background

**Fig. 1:**
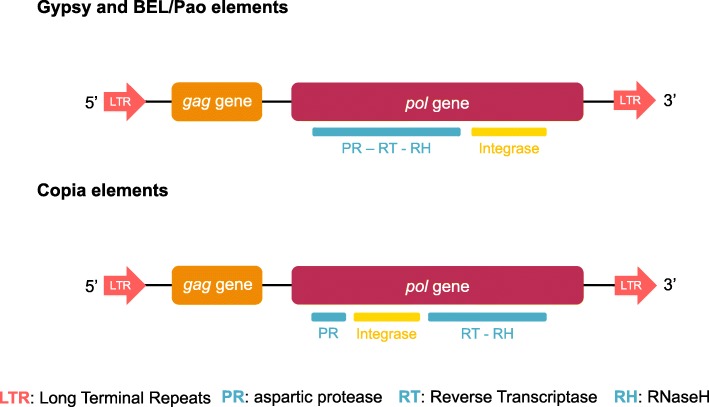
Schematic structure of LTR-retrotransposons elements. The long terminal direct repeats, flanking the elements, are represented by oriented red arrows and the two classical open reading frames by the two large rectangles. In the *pol* gene, the relative position of the domains that encode all the proteins required for transposition are detailed, in particular the integrase position

Transposable elements (TEs) are present in all eukaryotic genomes and play an important role in evolution by creating genetic variation through their mobility [1]. Retrotransposons transpose using a RNA intermediate and, because of their replication mechanism (“copy and paste”), are generally present in large numbers. They display a successful evolutionary history as shown by their broad phylogenetic distribution [2, 3]. Among the five major orders of retrotransposons [4], elements with Long Terminal Repeats (LTRs) are flanked by large (usually between 100 and 500 bp long) direct repeated sequences. These LTRs encompass the promoter and regulatory regions and also play a major role in the transposition cycle. So, LTR-retrotransposons are related to retrovirus [5, 6]. They usually encode two genes (*gag* and *pol*) in a single or two open reading frames [7] the *gag* gene encodes proteins involved in the formation of the virus-like particles; and the *pol* gene encodes various protein domains involved in the transposition mechanism, like a protease, an integrase, a reverse transcriptase (RT) and a RNaseH (Fig. 1). These last two domains are always consecutive and adjacent. Therefore they are typically grouped into a single sequence (RT/RNaseH) that is conventionally used to reconstruct LTR-retrotransposon phylogenies [8].

Within LTR-retrotransposons, three superfamilies, Copia, BEL/Pao and Gypsy, have been characterised to date [9]. All LTR-retrotransposons remain quite similar in terms of structural features, sequences and replication mechanisms. However, those different superfamilies form distinct groups based on the phylogeny of their most conserved domains [5–7]. Gypsy and BEL/Pao elements also differ from Copia elements by the position of the integrase in *pol* gene (Fig. 1). However it should be noticed that some exceptions exist: the unusual Gmr1 clade regroups Gypsy elements whose integrase domain lies upstream of the reverse transcriptase domain, an arrangement that is characteristic of *Copia* elements [10]. TEs characteristics greatly impact their dynamics and success in the genomes. For example, while LTR-retrotransposons make up the largest proportion of plant TEs, they are less predominant in animals and absent in prokaryotes. Different measures can be used to quantify the abundance of TEs: (i) the number of copies within a genome; (ii) the genomic proportions (percentage of the genome in base pair); (iii) the diversity (i.e. the number of different families or clades from one superfamily in a given species or taxon); (iv) the distribution (i.e., repartition of each family, clades or superfamily among different host species or phyla). Herein we define a TE family (an element) as a cluster of related TE copies within a given genome, according to the 80–80-80 rule which has been proposed as a means to identify copies from the same TE family: two TE copies may be considered belonging to the same family if they are aligned with 80% identity, over at least 80 bp and 80% of their respective lengths [4]. In addition, a TE clade refers to a monophyletic group of families present in different host species. Finally, phylogenetically related clades may be grouped in a lineage (for example, A-clade, B-clade and C-clade of the Mag lineage of Gypsy elements [11]).

Copia and Gypsy elements are widely distributed among genomes of plants, fungi and animals but no BEL/Pao elements have been identified in plants, fungi or mammals so far [11] In metazoans, the three superfamilies display uneven relative abundances among genomes that greatly depend on both the element type and the host taxon considered. Previous comparative analyses in insects, nematodes and chordates [12], crustaceans [13], fungi [14] and more specifically Pezizomycotina [8], revealed that Gypsy and Copia elements display different distribution, representativeness and diversity. The Gypsy elements seem clearly the most abundant LTR-retrotransposon superfamily, found in almost all tested species, with a large number of copies in the available genomes. Moreover, they appear highly diversified with numerous families and clades. In contrast, Copia elements are typically less frequently detected [13] and consequently appear much scarcer and absent in one third of the metazoan genomes analysed [12]. Indeed, they usually display a low copy number [8] which is significantly lower than the Gypsy elements in most cases [12, 14]. They have a low diversity in terms of both family and clade numbers. In crustaceans, a great majority of Copia families belong to a single dominant clade, GalEa, which is widely distributed among metazoans [13, 15]. An equivalent pattern is found in Pezizomycotina in which only two clades, GalEa and Funco1, account together for more than 80% of both detected sequences and families of Copia, whereas other families are scattered in a series of small clades [8]. These patterns suggest that Copia and Gypsy retrotransposons likely present two different dynamics [13]. Gypsy elements are frequent and diverse. They could simply follow a Red Queen dynamics [16] in which elements constantly transpose and evolve to escape the host’s regulatory mechanisms. Even if Copia elements could also evolve through an “arms race”, their dynamics most likely follow a “Domino Day spreading” model [8, 13] in which only few clades are maintained due to amplification bursts in particular taxonomic groups, suggesting the influence of additional evolutionary forces. However, even if comparative studies of Copia and Gypsy in Opisthokonta [8, 12–14] converge, in each case the findings remain limited either by the method used to detect TEs, the diversity of species analysed or by the number of parameter used to describe TEs. The PCR approach used in the study of crustaceans greatly limits the number of families detected and does not allow the estimation of the copy numbers. In Pezizomycotina, only Copia elements were analysed. In other studies, phylogenetic analyses are missing. A fully integrative study over a large set of species within a large phylum appears therefore still necessary to confirm the consistency of the different dynamic models of the Copia and Gypsy elements.

Regarding the BEL/Pao superfamily, seven clades are now well described, namely BEL, Pao, Sinbad, Suzu, Tas, Flow and Dan [12, 17]. The two last ones have been characterised recently through a large comprehensive genomic analysis carried out on 62 genomes of metazoans [12]. More precisely, the Dan clade originated from the split of the former Pao clade in two separate clades (Pao and Dan). Such results show that studying new host phyla can substantially improve the knowledge of LTR-retrotransposon diversity. This study especially demonstrated that BEL/Pao seems to be the second most abundant superfamily of LTR-retrotransposons in metazoans considering its frequency in species and copy number in the genome [12]. However, the taxonomic diversity of host species studied appears limited and mainly restricted to insects (especially drosophila), nematodes and chordates, which circumvents other large phyla and may bias our predictions in terms of evolutionary success and species occurrences. Thus, more effort is still needed to investigate BEL/Pao elements in a new large host phylum to get a better understanding of their pattern of abundance and diversity.

Recently, several taxa of ecological and evolutionary significance began to be investigated for the presence of transposable elements in the genome, but many of them still have received little attention. Molluscs are a great model to investigate LTR-retrotransposons within a phylum. With more than 100,000 living species, molluscs are the second largest metazoan phylum after arthropods [18] and display a large diversity of species inhabiting a various set of environments (freshwater, marine and terrestrial ecosystems). They are one of the most diverse groups of animals with eight classes of living molluscs. Their body morphology is incredibly variable, ranging from minute wormlike interstitial animals to giant squids and from microscopic snails to giant clams. Despite their incredible diversity, genomes of molluscs have received very little attention in the past regarding transposable elements. Few DIRS1-like retrotransposons were detected in *Aplysia californica* and *Lottia gigantea* genomes [2]. Some recent studies of mollusc TEs refer to transposons (MITE Pearl in *Crassostrea virginica* [19], Tc1/mariner in *Littorina saxatilis* [20]) and novel superfamilies of SINE elements in gastropods and bivalves [21, 22]. Considering LTR-retrotransposons, an active Gypsy element, Steamer, has been detected in leukemic cells of *Mya arenaria* [23] and thereafter observed in divers bivalves [24]. Few new families from *A. californica*, *Crassostrea gigas*, *L. gigantea* were also registered in Repbase [25]. The fraction of TEs estimated in mollusc genomes varies between 2 and 8% [26–30]. Among the TE-derived sequences identified in the flat oyster *Ostrea edulis,* only a small part correspond to LTR-retrotransposons (22 on 1226 fragments) [31]. Nine complete genomes distributed among the three major classes of molluscs (bivalves, gastropods and cephalopods) have been now published: the oyster *Crassostrea gigas* [30], the Mediterranean mussel *Mytilus galloprovincialis* [32], the pearl oyster *Pinctada fucata* [29], the sea hare *A. californica* (Broad Institute), the Ramshorn snail *Biomphalaria glabrata* [33], the Tribble’s cone *Conus tribblei* [34], the owl limpet *L. gigantea* [26], the great pond snail *Lymnaea stagnalis*, and the California two-spot octopus *Octopus bimaculoides* [27]. In addition, partial genome or transcriptome sequences have been reported for a larger number of mollusc species [28]. We took advantage of this available genomic and transcriptomic data to carry out the first large-scale comparative genomic analysis of LTR-retrotransposons in molluscs and to thoroughly investigate the phylogenetic relationships between the different clades of Copia, BEL/Pao and Gypsy elements in this phylum.

## Results

### Variable abundance of LTR-retrotransposon superfamilies in mollusc genomes

The nine genomes of mollusc screened for the three superfamilies of LTR-retrotransposons were those available in January 2017 from the Genome Online Database (GOLD, https://gold.jgi.doe.gov) and sufficiently well-assembled (see Additional file 1 for metrics). For example, we discarded draft genomes of both *Dreissena polymorpha* and *Corbicula fluminea* for which the length of the assembled genome does not mirror the real genome size. Genome size included in our study ranged from 0.36 Gb for *L. gigantea* to 2.34 Gb for *O. bimaculoides.*

**Fig. 2:**
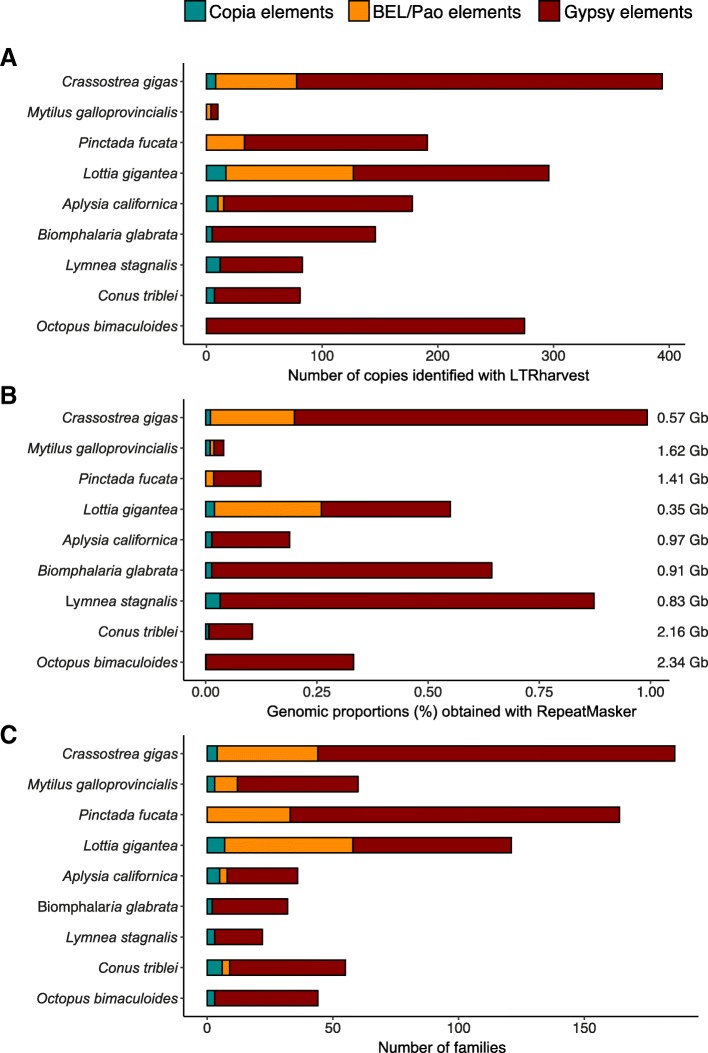
Relative LTR-retrotransposon’s content of each host genome. The horizontal axis indicates the abundance of Copia (turquoise), BEL/Pao (orange) and Gypsy (maroon) superfamilies in each genome estimated from **a**) the number of copies obtained with LTRharvest; **b**) the genomic proportion (%) obtained with RepeatMasker and **c**) the number of distinct families/elements

Using the software LTRharvest [35] we identified de novo 1637 copies that can be assigned to a LTR-retrotransposon superfamily, including 49 Copia, 217 BEL/Pao and 1371 Gypsy. Overall, the number of copies detected was low as it varied from six for *M. galloprovincialis* to 393 for *C. gigas* with a mean value of 182 copies per genome (Fig. 2a). Gypsy elements occurred in all genomes, Copia elements were found in six genomes and BEL/Pao elements in only five. In our genome set, only *C. gigas*, *A. californica* and *L. gigantea* seem to possess the three kinds of well conserved retrotransposons. The relative abundance of the three superfamilies is highly variable between species. Gypsy elements accounted for 100% (275 copies in *O. bimaculoides*) to 57% (169 copies in *L. gigantea*) of all copies detected with LTRharvest (Fig.e 2a). When found, BEL/Pao elements were the second most represented superfamily (up to 37% in *L. gigantea*). At last, Copia elements were rare and at the highest, accounted for 15% of the LTR-retrotransposons (12 copies in *L. stagnalis*).

Because the identification procedure of TEs with LTRharvest purposely excludes small deleted or altered copies, we secondly searched for LTR-retrotransposons copies using RepeatMasker and a database including all curated LTR-retrotransposon sequences previously identified in the nine genomes (see Database2 in Methods). With this approach, Copia elements were detected in the genomes of two other bivalves: *M. galloprovincialis* and *O. bimaculoides*; and additional BEL/Pao elements were found in the genome of *C. tribblei* (Fig. 2b and c). Subsequently, we estimated the genomic proportions of the different superfamilies considering all sequences identified with RepeatMasker (Fig. 2b). LTR-retrotransposons still represent only a small part of the mollusc genomes from 0.02% in *M. galloprovincialis* to 0.99% at most in *C. gigas*. Both results obtained with RepeatMasker and LTRharvest are consistent regarding the relative abundance of the different LTR-retrotransposons. These results are consistent with the estimated abundance of TEs through reads analysis using the dnaPipeTE software [36]. Indeed, in each species analysed, the relative abundance of the different LTR-retrotransposons is comparable to the one obtained with RepeatMasker on assembled genomes (Additional file 2). Only two particular discrepancies can be pointed out. For *B. glabrata*, several reads were annotated as BEL/Pao sequences whereas no element of this superfamily has been detected in the assembled genome. In the same way, for *A. californica*, numerous reads appear related to BEL/Pao whereas only three copies were detected in the genome. We can also observe some differences between the two approaches concerning the total abundance of LTR-retrotransposons, which appears sometimes higher using reads analysis compared to assembled genome mining. Nevertheless, whatever the approach used, the LTR-retrotransposons part in mollusc genomes appears small (less than 1%). We obtained almost the same genomic proportions (from × 1 to × 1.5) for *C. gigas*, *B. glabrata*, *L. stagnalis* and *O. bimaculoides*. But for *C. tribblei,* LTR-retrotransposons appear four times more abundant based on reads mapping.

In order to see if the comparative abundance is also mirrored in the diversity of elements, we characterised the different families (Fig. 2c). We reported 724 families, which were defined according to four different approaches (Additional file 3). The clustering of copies obtained with LTRharvest allowed us to define 249 clusters, which are hereby considered as TE families. Most of them contained few sequences; the largest family carried 23 sequences and was found in the genome of *A. californica* (Gypsy-1_AC). LTRharvest also revealed orphan sequences that did not cluster with any other one. Among them, the 239 that harbored a translatable RT/RNaseH domain were considered as additional families. Twenty supplementary families were defined using referenced sequences registered in Repbase [25] but not found in our study (due to the absence of LTR or RT/RNaseH domain). Finally, 216 other families were defined with sequences obtained with RepeatMasker (see Methods for details). The use of RepeatMasker highly increased the number of families for three species: *M. galloprovincialis, P. fucata* and *C. tribblei (*respectively 58 over 63, 37 over 65 and 76 over 131). In these three species the diversity of families was much higher than its expectation from the number of copies only detected with LTRharvest (Fig. 2a vs 1C). Overall, the number of families ranged from 20 for *L. stagnalis* to 164 for *C. gigas*.

In conclusion, for all parameters considered (i.e., number of copies, coverage of the genome or number of families), Mollusc LTR-retrotransposons are composed of approximately 83% of Gypsy, 13% of BEL/Pao and only 4% of Copia considering all gathered molluscs.

### Phylogenetic relationships among LTR-retrotransposon families

The phylogenetic relationships of LTR-retrotransposons found in molluscs were reconstructed to infer a classification of these elements and estimate their diversity. To this end, we performed phylogenetic analyses of elements that are representative of the newly identified families and of elements that are representative of the reference clades previously reported in literature (see Additional file 1 for details). As nine genomes may be too weak to have a holistic view of the diversity, we added numerous mollusc LTR-retrotransposons sequences detected by tBLASTn similarity-searches, mainly from transcriptomic data (Additional file 4). We defined clades based on the two following criteria: a clade includes sequences from at least two distinct species; and a clade is supported by a bootstrap value higher than 70 [8]. The clades, including those not appearing in the phylogenetic tree, are given in Additional files 3 and 4, and the total number of elements identified by clade is given in Table 1.

**Table 1:** Number of families from each clade found in the complete genome sequences or from retrotransposon sequences from databases

Superfamily/clade	Genome^a^		Database^b^
Copia			
GalEa	15	(183)	57
Hydra	6	(125)	37
CoMol	1	(185)	3
BEL/Pao			
Sparrow	70	(425)	60
Sinbad	24	(241)	32
Surcouf	8	(28)	15
TAS	16	(91)	76
Suzu	12	(127)	19
Flow	2	(9)	12
Dan	0	(0)	5
Gypsy			
C-clade	111	(3021)	114
MolGy1	73	(539)	66
AB-clade	32	(3031)	43
MolGy2	149	(2302)	83
MolGy3	38	(264)	20
MolGy4	9	(916)	17
MolGy5	9	(5111)	17
CsRN1	24	(3115)	31
MolGy6	41	(302)	20
MolGy12	9	(284)	16
Cigr-1	12	(1476)	3
MolGy7	3	(85)	7
MolGy9	0	(0)	10
MolGy13	8	(31)	0
MolGy8	2	(780)	5
MolGy10	3	(43)	4
MolGy11	5	(53)	2
MolGy16	7	(1029)	1
MolGy14	3	(32)	2
MolGy15	0	(0)	4
Tor2	0	(0)	4

^a^In the nine studied species, the number of copies is given in brackets

^b^In 46 species

**Fig. 3:**
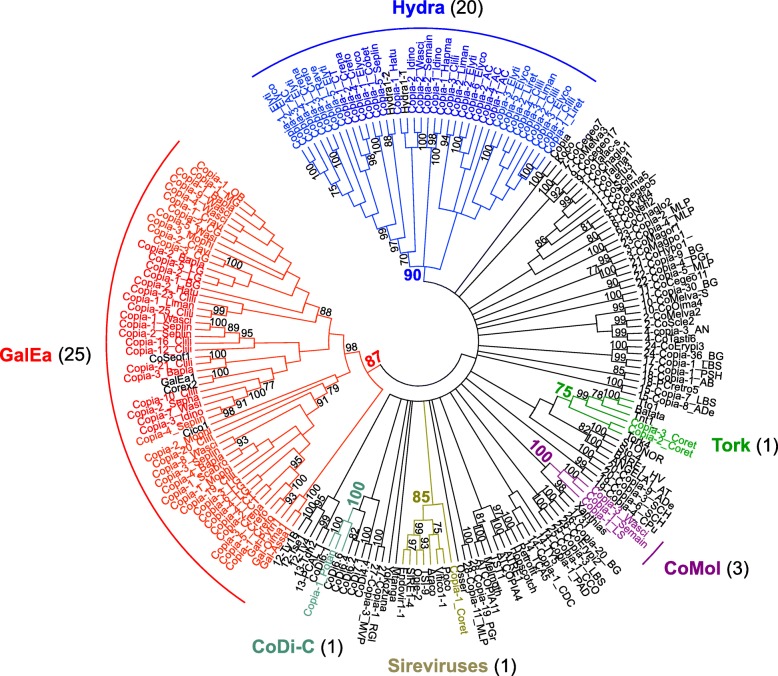
Phylogenetic relationships of Copia retrotransposons. The tree is based on Neighbor-Joining analysis of RT/RNaseH domain amino acid sequences. The Copia families from molluscs are indicated in color and arc of different colors indicate major clades. The number of mollusc species covered by each clade in the phylogeny is given into brackets. Node statistical support values (> 70%) come from non-parametric bootstrapping using 100 replicates

For Copia elements, the resulting tree includes 192 sequences of which 93 are from mollusc (Fig. 3). It revealed that the GalEa and Hydra [9] clades highly dominate Copia content as they account together for 96% of the new characterised elements (72 and 43 families respectively, Table 1). Beside these two major clades, we defined a new small clade named CoMol (for Copia of Molluscs) which contains only the Copia-1_LS family from the genome of *L. stagnalis* (11 copies) and three families from transcriptomes of cephalopods (*Sepia pharaonis*, *Sepia maindroni*, *Watasenia scintillans*). Curiously, the four remaining Copia sequences appear to group in algae or plants clades: the only sequence found in the gastropod *Potamopyrgus antipodarum* belongs to the CoDi-C clade [37] and the sequences from the gastropod *Colubraria reticulata* belong to the Sireviruses or Tork clades. Even if we cannot exclude that these sequences could have originated from horizontal transfers, three arguments lead us to suspect contamination in these two species due to their environment and/or diet: (i) it concerns only very few sequences which only come from transcriptomic data, (ii) the sequences of each of these clades are detected in a unique mollusc species, (iii) no other Copia elements were detected in these transcriptomic data. Thus, we did not consider these potentially artefactual clades in the rest of the study.

**Fig. 4:**
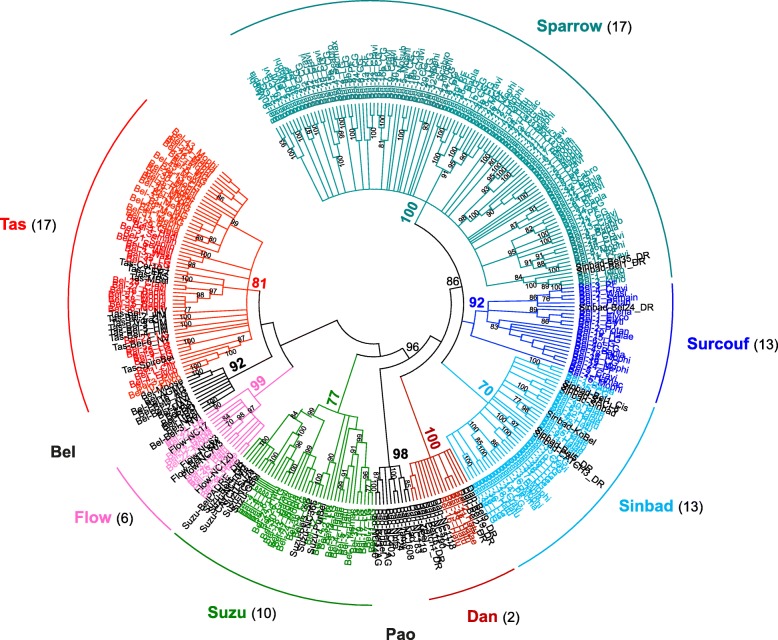
Phylogenetic relationships of BEL/Pao retrotransposons. The tree is based on Neighbor-Joining analysis of RT/RNaseH domain amino acid sequences. The BEL/Pao families from mollusc are indicated in color and arc of different colors indicate major clades. The number of mollusc species covered by each clade in the phylogeny is given into brackets. Node statistical support values (> 70%) come from non-parametric bootstrapping using 100 replicates

Using the same approach, we built a phylogenetic tree for BEL/Pao elements which includes 316 sequences of which 248 are from mollusc (Fig. 4 and Table 1). None of the BEL/Pao sequences identified belonged to the BEL or the Pao clades, after which the BEL/Pao superfamily was originally named. However, we identified elements that belong to the five other known clades: Dan, Flow, Tas, Suzu and Sinbad. This is a little surprising for Dan and Flow, as elements from these two clades are usually rare. The Dan clade was previously only described from the zebrafish *Danio rerio*. With this study, five elements were also found in the gastropods *Haliotis laevigata* and *Haliotis tuberculata*. Similarly, the Flow clade had originally been defined from only five families found in two cnidarians and one planarian [12]. Here, we found 14 additional families in three mussels (*Bathymodiolus platifrons*, *Modiolus philippinarum*, *Mytilus californianus*) and three gastropods (*H. laevigata, H. tuberculata*, *L. gigantea*). With 92 elements, the Tas clade appears to be the second best represented clade of BEL/Pao in molluscs, whereas the Suzu clade is less represented (31 elements). Our phylogenetic analysis also reveals a major difference compared to the previous studies. Here we show that elements inside the Sinbad group can be further classified into three separate clades (blue clades in Fig. 4). Among the 209 families clustered into this lineage, only 56 belonged to the sensu stricto Sinbad clade. Other elements fell into two seemingly mollusc-specific clades: a well-diversified clade presently named Sparrow containing most of the molluscs BEL/Pao elements (153 families) and a smaller clade named Surcouf containing the remaining elements (23 families). These three closely related clades group in what we choose to call the Sailor lineage (bootstrap value of 86) that represents more than two third of the BEL/Pao elements in molluscs.

**Fig. 5:**
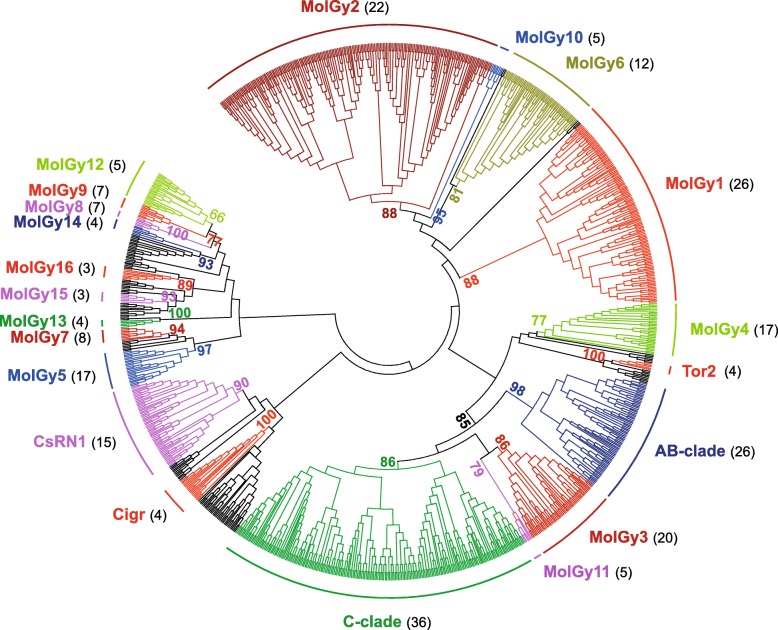
Phylogenetic relationships of Gypsy retrotransposons. The tree is based on Neighbor-Joining analysis of RT/RNaseH domain amino acid sequences. The Gypsy families from mollusc are indicated in color and arc of different colors indicate major clades. The number of mollusc species covered by each clade in the phylogeny is given into brackets. Node statistical support values (> 70%) come from non-parametric bootstrapping using 100 replicates

For the Gypsy superfamily, the reconstructed tree includes 1060 sequences of which 989 are from molluscs and reveals 21 clades in molluscs (Fig. 5 and Table 1). A simplified representation is available and shows mollusc elements of a same clade as compressed subtrees (Additional file 5). It allows to better distinguish the reference Gypsy elements and so, to determine whether a clade has been previously reported [9]. Only six known clades of Gypsy are retrieved in molluscs. The C-clade, which includes the SURL element [38], is one of the most dominant since it encompasses more than 220 mollusc elements. It is followed by the A-clade and B-clade which represent together 75 elements. In fact, using CFG1, Gulliver, Hydra2–1 and Mag elements as reference, we were unable to clearly distinguish these two clades. The CsRN1 clade [39], characterised by the elements CsRN1 and Kabuki, is also fairly well represented in molluscs with more than 50 elements. The last two clades Cigr-1 [40] and Tor2 [41] are smaller with less than 15 elements. The 16 remaining clusters likely correspond to new mollusc-specific Gypsy clades. These clades have been named MolGy (**Mol**lusc **Gy**psy) and numbered from 1 to 16 following the decreasing number of occurrences in species. Note that an exception has been made for the clade MolGy12 which despite a low bootstrap value of only 66, has been defined as a new clade grouping 25 sequences from five distinct mollusc species. MolGy3 and MolGy11, group with the A-clade, B-clade and C-clade, suggesting that they would be part of the Mag lineage (bootstrap value of 85). Then, this lineage encompasses more than a third of the Gypsy elements detected in molluscs (Fig. 5 and Table 1). Other MolGy clades are scattered in the tree and do not group with known clades. MolGy1 and MolGy2 are particularly large clades (139 and 232 elements, respectively), as large as the C-clade. The four clades, MolGy3 to MolGy6, also possess a fairly high number of elements (from 26 to 61) and appear as important and diverse as the AB-clade and CsRN1 clades. The remaining MolGy clades (MolGy12 apart because of his particular status) seem small with less than 10 elements detected.

### Distribution of clades in mollusc species

**Fig. 6:**
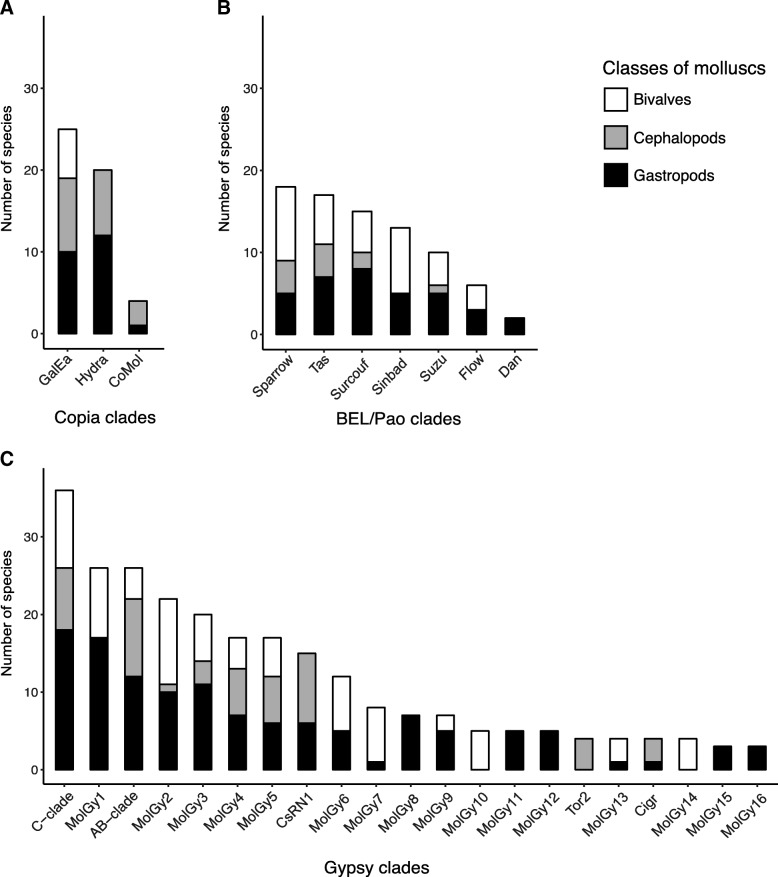
Distribution of LTR-retrotransposon clades among molluscs. The number of species in which each clade occurs is given for the 3 studied classes of molluscs. **a**) Copia clades, **b**) BEL/Pao clades, **c**) Gypsy clades

As phylogenetic trees revealed major and minor clades containing more or less elements, we checked whether this feature could also be true in terms of distribution among host species. The database sequences include 15 bivalves, 21 gastropods and 11 cephalopods. It should be noticed that for several of them, the sequences available may be relatively limited, thus an absence of detection do not certify that a clade is not present. The number of species and taxonomic classes associated with each clade is shown in the Fig. 6 and the lists of species included in Additional files 3 and 4.

The three superfamilies appear to have different patterns of distribution in molluscs. Both Copia and BEL/Pao elements are concentrated in few clades well widespread among the studied species, even if Copia clades seem quite more frequent than BEL/Pao ones (Fig. 6a and b). Copia elements were detected in 28 species. Both GalEa and Hydra clades are well distributed, respectively in 25 and 20 species. They are especially found in two-thirds of cephalopods studied. On the contrary, no element from the Hydra clade was found in the 18 bivalve species, neither in genomes nor in databases. BEL/Pao elements were detected in 26 species. Although the Suzu clade seems more restricted (only ten species), the five major clades are present in a wide range of host organisms, up to 18 species for Sparrow. The Sailor lineage is then widely distributed in almost half of the species and highly dominates BEL/Pao distribution. Nevertheless, Sinbad elements have not yet been detected in cephalopods, although this clade is present in 13 mollusc species.

Gypsy elements are by far the most widespread in molluscs, being detected in 51 of the 56 species in which LTR-retrotransposons have been identified (Additional file 3). This suggests that almost all molluscs (at least for the three classes studied) possess Gypsy elements. Gypsy clades can be sorted in two types (Fig. 6c). The nine most diverse clades (Table 1) were also the most common ones and were distributed in 12 (MolGy6) to 36 species (C-clade). The other clades were rarer and were detected at the most in eight species (MolGy7). As expected, most of the common clades are spread over the three mollusc classes. However, MolGy1 (26 species) and MolGy6 are not found in cephalopods. Conversely, the CsRN1 clade is very well represented in cephalopods (9 on 12 species) but is not observed in bivalves.

### Clade proportions in genomes

**Fig. 7:**
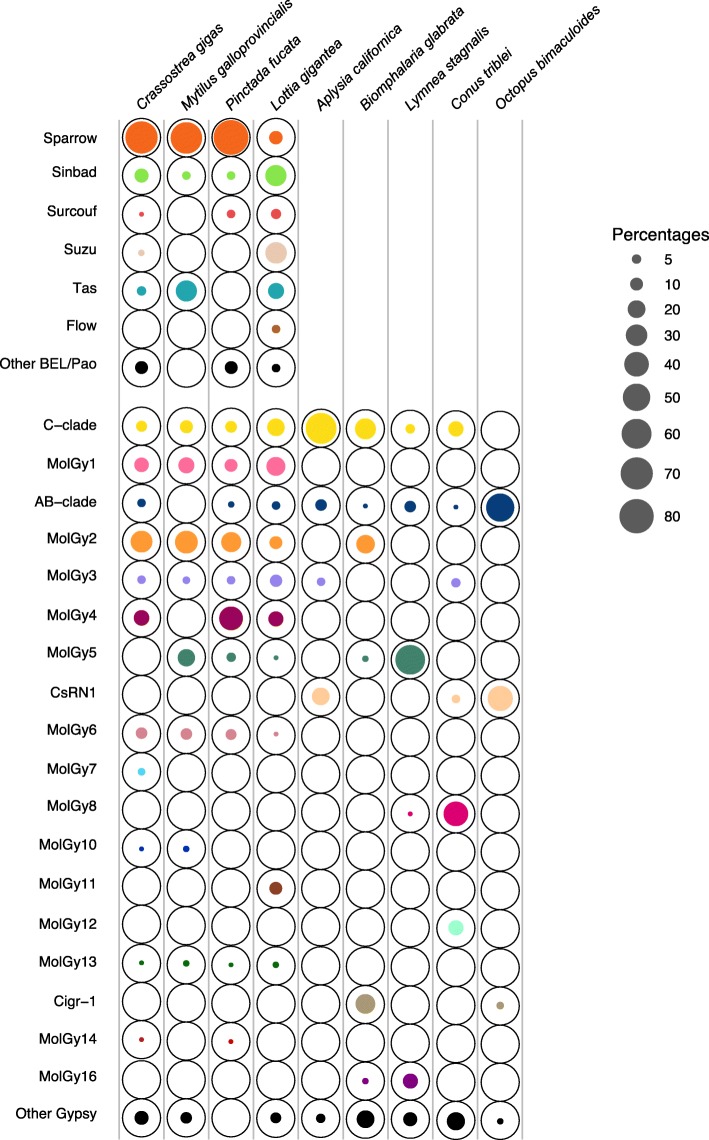
Relative proportion of BEL/Pao and Gypsy clades in the genomes of the nine mollusc species. For each genome (column), the bubble chart shows the relative distribution of the clades considering the length (in base pairs) of all sequences obtained with RepeatMasker. Each black circle indicates 100% for a given superfamily and the surface covered by the colored solid circle indicates the relative of proportion the given clade for a given superfamily in each genome. “Other BEL/Pao” and “other Gypsy” rows refer to LTR-retrotransposon sequences that could not be included in any major clade

In addition to the distribution of the different clades among species, we were interested in their relative proportions in the genomes presented in Fig. 7. Lonesome elements (not linked to a clade and grouped as “other”) represent a maximum of 10% of BEL/Pao elements (*C. gigas* and *P. fucata*). For Gypsy elements, even if they appear negligible in *P. fucata* and *O. bimaculoides*, they represent at least 5% in other genomes and up to 20% in the gastropods *B. glabrata* and *C. tribblei*. Such high percentages can be explained by the great diversity of Gypsy elements. However, as they do not constitute a clade, these groups were not considered in the rest of the analysis.

The analysis of the BEL/Pao clades included only the four genomes which possess more than ten families. The three bivalves show a similar pattern in which Sparrow and Sinbad clades together account for at least 70% of the BEL/Pao elements, the Sparrow clade being largely dominant. A very different pattern is observed for the limpet *L. gigantea* in which six clades out of seven (Dan except) are well represented. This is all the more surprising as the other gastropods are almost lacking BEL/Pao elements. In this species, the three clades from the Sailor lineage represent only 46% overall; while the Suzu clade, seldom observed in other genomes, reaches 30%.

Regarding the Gypsy elements, on the one hand mollusc genomes display very different patterns that can however be grouped in three categories: (i) a dominant clade can clearly make up the majority of classified Gypsy elements, such as in *A. californica L. stagnalis* and *C. tribblei*; (ii) few major clades can be observed in similar proportions in *B. glabrata* or in *O. bimaculoides*; (iii) about half of the known Gypsy clades from mollusc can be well represented as in the three bivalves and in *L. gigantea*. On the other hand, we wondered if the clades supported by a large number of families are also strongly represented in diverse genomes. It seems to be somewhat the case for the three major clades (C-clade, MolGy2 and to a lesser extend MolGy1). Among the moderately diverse clades, four are well represented in at least one or two species (AB-clade, MolGy4, MolGy5 and CsRN1); whereas, the MolGy3 and MolGy6 clades never represent more than 9% of the Gypsy elements. The remaining clades, which typically showed a low diversity or distribution in molluscs, reveal two opposite dispersals. Some of them, such as Cigr-1 or MolGy8, represent a large proportion in a particular genome; whereas the others, such as MolGy7 or Moly13, always remain below 3%.

So, considering all the superfamilies, some species display a rich and diverse distribution of LTR-retrotransposons in their genomes, notably *L. gigantea* and, to a lesser extends, the bivalves. Moreover, the 3 species of bivalves show very similar patterns in term of variety and proportion of the different clades for both BEL/Pao and Gypsy elements.

## Discussion

### Studying LTR-retrotransposons within a large host phylum

Molluscs are a great model to investigate the distribution and the diversity of LTR-retrotransposons and thus to refine the dynamic models. Even if the number of genomes assembled to date may still seems paltry considering the diversity of this phylum, the current search, although not exhaustive, may give a nice (if not a first) idea of LTR-retrotransposons distribution among molluscs. Moreover, the sequencing of non-model species is increasing and accelerating, and since the beginning of our analysis 3 more genomes have been published: *B. platifrons* and *M. philippinarum* [42], *Crassostrea virginica* [43]. Even if the genomes provide essential information, they remain often limited in number to properly describe LTR-retrotransposons in molluscs. Thus, increasing the number of considered species using sequences from databases is helpful to complete and improve the diversity of families and clades. This is also essential to assess clades distribution, because the largest diversity of potential hosts is needed to infer that a clade is missing in a given taxonomic level. A restriction in regard of such additional data is that most of the time the depth of the genomic or transcriptomic sequencing remains unknown. Nevertheless, some reference transcriptomes are also available (*Colubraria reticulate* [44], *Clione limacine* [45], *Crepidula navicella* [46], *Haliotis tuberculate* [47]). It is however unfortunate that no genomic or transcriptomic information is available about the less diverse classes (Polyplacophora and Scaphopoda, for example).

Scanning diverse genomic and transcriptomic databases led us to described 812 elements in addition to those obtained with genomes (52.5% of the families). This provides crucial information about distribution and families diversity. However, these elements do not seem essential to assess most clade diversity, because the nine genomes alone were sufficient to get most of the LTR-retrotransposon clades that we found overall. This result also underlines that the detection strategy based on LTRharvest is efficient. Indeed, we did not find 17 of the 152 mollusc LTR-retrotransposons given in Repbase, but on the other hand we have characterised 204 new families in the three species considered.

### Contrasting patterns of Copia and gypsy elements

Abundance and diversity of Gypsy and Copia elements were consistent with two different dynamics. As in almost all metazoan genomes studied until now, Gypsy elements are the most abundant superfamily in all mollusc genomes. On the other hand, the overall distribution and scarcity of Copia elements among molluscs, crustaceans or Pezizomycotina appear similar. For metazoans, only 8 clades of Copia elements are referenced in the Gypsy Database against 17 clades for Gypsy elements. In molluscs, almost all Copia elements belong to only the GalEa and Hydra clades. These results are consistent with those obtained in crustaceans where the GalEa clade appears also as the predominant one [13]. However, considering the prevalence of GalEa elements among Copia retrotransposons, this study is more similar to the one obtained in Pezizomycotina in which two clades (GalEa and FunCo1) were also strongly prevalent [8]. Note that in addition to the major clades only one poorly diversified clade has been described in Mollusca against 8 in Pezizomycotina. But this phylum is larger and more diverse than Mollusca for which data are only available for the three most important classes. Thus, it remains possible that the enrichment of our genomic dataset with new published genomes, especially from other mollusc classes, could allow us to discover additional small clades of Copia elements. Within crustaceans, the distribution of TE clades among species appears highly related to the host phylogeny [13]. But, this inference could not be assessed here as too few classes of molluscs are represented by available sequences. To a lesser extent, scarcity of Copia compared to Gypsy elements is supported by analyses of *Drosophila* genomes in which Copia elements turn out to be clearly rarer in number of copies, less diverse in number of families and clades, and correspond to a smaller proportion of the genomes [48]. In the end, the consistency of the results obtained in distant taxa clearly show that, no matter the phylogenetic group or the taxonomic level that we look at (*Drosophila*, Crustacea, Mollusca or Pezizomycotina), the two opposite patterns found for Copia and Gypsy elements confirm different dynamics. Copia elements may follow the “Domino Day spreading” dynamics already extensively described [8, 13], whereas Gypsy elements are more likely to display a “Red Queen hypothesis” dynamics [16]. A small part of this difference could be explained by the influence of horizontal transfers, which could promote the element diversity. For example, numerous horizontal transfers of Gypsy elements have been reported between bivalve species [24].

### Intermediate representativeness of BEL/Pao elements

BEL/Pao elements were the second most abundant superfamily in terms of copy number, number of families and clade diversity. These results are consistent with previous conclusions in which relative abundances of LTR-retrotransposons and DIRS-like retrotransposons show that BEL/Pao elements are the second superfamily in representativeness, after Gypsy elements [12]. At a genus scale, analysis of 20 Drosophila genomes also reveals that BEL/Pao elements, present in all species, are second in terms of number of families (192 families, compared to 66 for Copia and 345 for Gypsy elements) and proportion in the genomes [48]. In the *Drosophila* genomes, LTR-retrotransposons are the most widely represented order of TEs, which may explain that the proportion of BEL/Pao elements is slightly higher than in molluscs. The presence of BEL/Pao in genomes seems to be phylum-dependent as different patterns can be observed in different taxa. This superfamily is found in a majority of species of chordates, insects and nematodes, but analyses of 11 mammal genomes revealed no BEL/Pao element [12]. Herein, we found elements of the BEL/Pao superfamily in about half of the genomes of molluscs, which is lower than what has been found in the three other phyla. At the clade level, results obtained in molluscs can be compared to those of this previous study [12] taking into account that around 60 species were analysed in each study (Additional file 6). No element from the BEL or Pao clades was detected in molluscs although they show the greatest number of copies and families (around 300 families) reported in other phyla. This apparent abundance of both BEL and Pao clades could be due to a sampling bias as many insect genomes were studied. Actually, these two clades are restricted to a few phyla and are almost entirely observed in insects where they predominate, sometimes exclusively as in *Drosophila*. We observed the same patterns of distribution among species for the Dan clade, and for the Tas and Sinbad clades which are described in quite the same number of species in both cases. Exceptions can be found for the Flow and Suzu clades, which seem to be more abundant in molluscs as we recorded them in twice as more host species and three times more families. Thanks to our study, we also extend our knowledge of BEL/Pao elements diversity with the description of two completely novel clades. The formerly recognised Sinbad clade is subdivided into three distinct and well supported clades. We kept the name Sinbad for the clade that contains the more referenced Sinbad elements and the original *Sinbad* element identified in *Schistosoma mansoni* [17]. For the two others, we propose new names also inspired from famous sailors: Sparrow and Surcouf. Apart from elements from molluscs, the Sparrow and the Surcouf clades contain only few reference sequences from the zebrafish, explaining why they have never been described before. Because of their intermediate patterns of distribution and diversity, it is trickier to infer the dynamics of BEL/Pao elements. Their dynamic appears clearly less efficient than that of Gypsy elements and thus could be closer to that of the Copia elements but made a little more efficient by recurrent intra-species diversifications with punctual emergences of several different new families inside a given species, as already described in *Drosophila* [48].

### LTR-retrotransposon clades among metazoans

If the three LTR-retrotransposon superfamilies are common in metazoans, their representativeness is not only given by their distribution within species but also by the number of phyla covered by each clade. Studies analysing LTR-retrotransposons clades often detailed whether they were widely distributed among phyla [12, 13]. In the same way, when a clade is depicted, the Gypsy database includes its distribution among host species and/or taxa.

Globally, the distribution of the Copia clades among metazoans appears heterogeneous. On the eight major clades from metazoans, six have only been described in arthropods, more precisely in winged insects (Copia [49]), Diptera (1731, Xanthias [48, 50]), or in a unique species (Tricopia, Mtanga, Humnum [9, 51]). Such monospecific clades would not have been considered in our study if we had encountered such a case for Copia elements. Considering that the Mollusca phylum is larger than the subclass of winged insects, we would have expected to find more small clades in molluscs. Interestingly both remaining Copia clades, GalEa and Hydra, seem to be absent in insects. GalEa elements were already observed in seven mollusc species and have a widespread distribution among metazoans (Crustacea, Chordata, Cnidaria, Ctenophora, Echinoderma, Hemichordata and Teleostei) [13]. Moreover, their presence in Pezizomycotina fungi [8] and red algae [13, 52] suggests that they were already present in the last common ancestor of Opisthokonta and are probably more ancient in eukaryotes, exception made of the hypothesis of multiple horizontal transfers. The Hydra clade was also observed in various phyla but to our knowledge they were only identified in few species: the cnidarian *Hydra magnipapillata*, the zebra fish *Danio rerio* [9] and the amphipod *Parhyale hawaiensis* [13]. Their large abundance in a fourth phylum such as molluscs strongly suggests that they potentially have a wide distribution that remains to be explored and compared with that of GalEa elements. Another question is about the distribution of less prevalent clades like CoMol. Considering that it was detected only in few molluscs, we can hypothesise that this clade recently emerged in this phylum. However, according to the dynamic model of Copia elements, we cannot exclude that such a clade may be well represented in another (or several) phyla.

The same question could have also arisen for small BEL/Pao clades, but our results showed that the Dan clade is not monospecific and that the Flow clade is present in at least 3 phyla. Pending analyses on other phyla, we can hypothesise that these clades will remain weakly represented in diverse phyla. Among the large clades, BEL and Pao were detected predominantly in insects, even if two BEL elements were depicted in a sponge [12]. Some insect species also harbor few Tas elements. However, the Tas, Suzu and Sinbad clades have been observed in diverse phyla from Porifera to Sauropsida [12]. We can thus presume that they are widely present in metazoans and it is therefore not surprising to see them in molluscs. Concerning the Sparrow and Surcouf clades, it is difficult to extrapolate their presence outside Mollusca. They might be a hallmark of BEL/Pao elements from molluscs. Conversely, these elements may have a wider distribution if previously assimilated to former clades, especially Sinbad, because of their rarity.

It is more difficult to consider the distribution of Gypsy clades among metazoans due to the great diversity of families and clades. Presumably, there must be numerous small clades restricted to each phylum. But the question of a wide distribution may be raised for the well diversified mollusc clades such as MolGy1 and MolGy2. While Cigr-1 previously constitutes a single-element-clade in the genome of *Ciona intestinalis* [11, 40] and Tor2 has been described in another tunicate *Oikopleura dioica* [41], both are present in molluscs and thus potentially in other phyla. The CsRN1 clade is present in the genomes of some protostome organisms [39]. Likewise, Mag is the most polyphyletic and widespread lineage and has been described in divers protostomes, echinoderms, insects and vertebrates [53–56]. It should come to no surprise that we detected them in molluscs since they are probably widespread across various phyla; and we suspect that the C-clade, previously based only on the echinoderm SURL element [38, 57] is even more widely distributed.

## Conclusions

In this study we carried out the first large-scale comparative genomic analysis of the LTR-retrotransposons in molluscs and identified 1709 families in total in 56 species. Whatever the parameter considered: copy number, proportion in the genomes and diversity of families or clades, Gypsy elements were unequivocally dominant and BEL/Pao elements were clearly the second-most abundant superfamily. Gypsy elements are present in almost all studied molluscs. BEL/Pao and Copia elements are only roughly equivalent in terms of number of host species, being detected in half of the considered species. According to their abundance, it seems that every time a new phylum is examined several new Gypsy clades are discovered, 16 in the present study. In addition to the 7 other clades already characterised in other taxa, molluscs reveal two new BEL/Pao clades (presently named Sparrow and Surcouf), which are largely represented in number of copies, families and hosts. We also defined a new Copia clade, CoMol, restricted to 4 families in 4 species. The two major GalEa and Hydra clades account together for 95% of the Copia elements. Our results are consistent with the “Domino Day spreading” dynamic model for Copia elements previously suggested on crustaceans and supported in Pezizomycotina, which relies on the fact that most of the presence of Copia elements in host taxa results from the evolutionary success of a few Copia clades.

## Methods

### Identification of retrotransposon copies with conserved LTR in mollusc genomes

The nine well-assembled mollusc genomes (see Additional file 1 for genomes information) available on January 2017 were downloaded from the National Center for Biotechnology Information [58] and the Okinawa Institute of Science and Technology (http://marinegenomics.oist.jp). For each genome, the analysis was performed individually but the approach and the different databases used were the same. We first isolated all potential LTR-retrotransposon sequences de novo using LTRharvest [35] based on the detection of two conserved LTRs and the following parameters: LTR length ranging from 80 to 1500 bp, distance between LTRs ranging from 2500 and 11,000 bp and sequence identity between LTRs higher than 80%. To discriminate LTR elements from artefactual sequences, we performed BLASTx similarity-searches on a custom Database1 comprising RT/RNaseH amino-acid sequences for 164 Copia, 122 BEL/Pao, and 116 Gypsy retrotransposons. This Database1 encompasses sequences from the Gypsy Database [11] appended with published sequences [8, 12, 13, 37]. This also allows us to classify sequences as Copia, BEL/Pao or Gypsy according to the best hit of blast results. This classification was further confirmed by phylogenetic analyses. Conserved sequences were then clean from microsatellite repetitions using tandem repeat finder [59].

The three resulting datasets of Copia, BEL/Pao and Gypsy nucleotide sequences (including LTR parts) from each genome were separately clustered using BLASTclust as in [8]. Copies belonging to a single cluster were then aligned with the E-INS-i iterative refinement configuration of MAFFT version 7 [60] and were manually analysed to define the boundaries of the LTRs. Moreover, sequences were manually curated to remove all copy-specific insertions larger than 20 bp. Individual copies may be indeed corrupted by insertion of various genomic sequences such as other transposable element parts that could strongly biased the estimation of abundance of each elements type among genomes using subsequent similarity-searches. We finally checked that all the curated copies from a cluster share at least 80% of identity over the whole DNA sequence, a threshold often used to define transposable element families [4]. Conversely, when elements from two clusters share more than 80% sequence identity, the clusters were merged into a single family. When a single sequence (=orphan sequence) was detected in a species, by default, we considered it as a representative of a distinct family. Such sequences had to possess a translatable RT/RNaseH domain in order to place them within phylogenies; otherwise they were excluded from the further analyses.

### Identification of additional LTR-retrotransposon sequences in mollusc genomes

For each species, the genomes were screened to recover all additional LTR-retrotransposon related sequences, including some putative false negatives from LTRharvest and shorter element derivatives. We used RepeatMasker version 4.0.6 [61] (options -nolow -no_is -pa 8 –frag 380,000 -div 20) and the unique custom Database2. Database2 contains all curated sequences from previous identified clusters in the 9 genomes and the LTR-retrotransposons describe in Repbase for *C. gigas*, *A. californica* and *L. gigantea* that were not previously recovered with LTRharvest. Isolated sequences (orphans) obtained with LTRharvest were not included in Database2 simply because they may contain contaminant fragments from other repeated sequence. As Repeat Masker is well known to subdivide genomic sequences, we used a customised script which concatenates, while integrating the central part, sequences of the same type (Copia, BEL/Pao or Gypsy) when distant of less than 500 bp (see Availability of Data and Materials section for the script).

The copies obtained with RepeatMasker were then assigned to the different families of LTR-retrotransposons using BLASTn (E-values 1e-10) and the custom Database3. Database3 corresponds to Database2 supplemented with families corresponding to orphan sequences previously discarded. In most cases, a copy was associated to a family of TEs that was previously defined in the analysed species. But in some cases, a copy can be related to family of TEs that has been defined in the genome of one of the other 8 species. These last correspond to copies for which no reference sequence could be detected by LTRharvest in the considered genome. For example, in the case where a species possesses Copia sequences in its genome but none of them have LTRharvest detectable LTRs, these Copia sequences are then recognised only through families from other molluscs. The copies that match with sequences from other species may define new families but could also correspond to highly altered sequences or false positives. As a consequence, only sequences with a recognizable and automatically translatable RT/RNaseH domain were retained and were then aligned and clustered to define new LTR-retrotransposons families.

Finally, families can be defined from 4 methods: clusters obtained with LTRharvest, orphans obtained with LTRharvest, referenced elements from Repbase but not identified with LTRharvest and sequences identified with RepeatMasker (Additional file 3). These final sequences include all possible families and were grouped in the Database4, made from Database 3 and sequences from new families defined with RepeatMasker. Database 4 was then used to reassign all sequences obtained with RepeatMasker to the different families of LTR-retrotransposons (BLASTn). Following this last procedure, only a few sequences were still not assigned to a given family and were thus gathered in an extra set, which was discarded from analyses.

### Reads analysis

Estimation of the abundance and the respective proportion of each LTR-retrotransposon family using reads were carried out using the dnaPipeTE software with default parameters [36]. For each species, reads that map on the corresponding mitochondrial genomes using the BWA software [62] were first discarded. DnaPipeTE were run on read subsamples ranging between a coverage of 0.01x and 0.5x in intervals of 0.05x (11 runs). For each of the 11 runs per species, we selected the subsample yielding the highest contig N50 in the assembly step of dnaPipeTE, as a measure of optimised read subsampling.

### Detection of LTR-retrotransposons in other databases

To identify LTR-retrotransposons more widely in Mollusca, we performed tBLASTn [59] analyses (e-values 1e-70, query cover > 80%, no filter) on genomic and transcriptomic databases (nr/nt, wgs, est., TSA) provided by NCBI. In the case of the Copia elements, which are relatively scarce, we used less stringent parameters (e-values 1e-40, query cover > 50%) and, sometimes, reconstructed chimeric elements (i.e. using overlapping of distinct sequences obtained from several copies which belong to a same family). Amino acid RT/RNaseH domains of elements that represent different clades of LTR-retrotransposons have been used as queries: 8 elements for Copia, 7 for BEL/Pao and 23 for Gypsy (see Additional file 1 for details). The identified amino acid sequences covering the RT/RNaseH domains were clustered and the largest sequence was chosen to represent each retrotransposon family. We used phylogenetic approaches to determine which clade these families belong to (see below). The remaining sequences were classified using similarity searches using BLAST on a database that includes classified mollusc elements. An element was then assigned to a clade when: (i) the five best hits correspond to referenced elements from this clade in the database; and (ii) the difference between the best E-values obtained and other reference elements is higher than 1e-10 [13].

### Phylogenetic analyses

Phylogenetic analyses were performed as in [8] on amino acid sequences corresponding to the RT/RNaseH domains of the newly characterised sequences, reference elements from Repbase or Gypsy Database, and previously identified Copia and BEL/Pao retrotransposons (Additional file 1). Boundaries of RT/RNaseH domains have been determined according to those define for RT 5′ part and RNaseH 3′ part of Copia, BEL/Pao and Gypsy multiple alignments defined in the Gypsy Database. DNA sequences were translated using a custom-made script and the longest representative of each family was selected. If a sequence appears corrupted by internal frameshifts, it was manually curated to reconstruct a chimerical protein sequence.

Multiple alignments of protein sequences were performed using MAFFT [60]. After a manual curation of the alignments, phylogenetic analyses were conducted using Neighbor Joining [63] and the pairwise deletion option of the MEGA5.2 software [64]. Using the Topali2.3 software [65], the best-fitted substitution model retained was the JTT model [66] with a gamma distribution. Support for individual groups was evaluated with non-parametric bootstrapping [67] using 100 replicates.

## Additional files

Additional file 1: List of genomes included in this study and previously referenced LTR-retrotransposons. The nine genomes of molluscs are listed together with the current URL and the accession numbers from which the genome sequences have been accessed, number of sequences of each genome included in our analysis, combined sequence sizes (in number of base pairs) and some metrics. Additionally we list all reference LTR-retrotransposons used from Repbase, the Gypsy Database, and previous publications; together with the species name they occur in. (XLSX 23 kb)

Additional file 2: Comparison of relative LTR-retrotransposon’s content estimated with RepeatMasker on assembled genome or dnaPipeTE using reads mapping. The horizontal axis indicates the abundance in kb of Copia (turquoise), BEL/Pao (orange) and Gypsy (maroon) superfamilies in each genome. (PDF 355 kb)

Additional file 3: Copy number and genomic proportions of the clades and families of LTR-retrotransposons detected in mollusc genomes. Copy numbers are given according to the estimation procedure. Additionally, we specify by which methods each family has been characterised. (XLSX 65 kb)

Additional file 4: Clades and families of LTR-retrotransposons detected in mollusc databases. The LTR-retrotransposons are listed together with the species name they occurred in, their accession number and the database type from which they were accessed. (XLSX 133 kb)

Additional file 5: Phylogenetic relationships among Gypsy clades. This tree is a simplified representation of Fig. 5, in which mollusc elements from a same clade are represented by compressed subtrees. All LTR-retrotransposon from a clade found in mollusc are depicted in color. The reference Gypsy elements and Gypsy clades previously reported in the Gypsy Database are in black. Node statistical support (> 65%) was obtained through non-parametric bootstrapping using 100 replicates. (PPTX 67 kb)

Additional file 6: Number of families, species and phyla for BEL/Pao superfamily. Comparison between results obtained in molluscs and those previously obtained in other phyla (de la Chaux and Wagner, 2011). (XLSX 11 kb)

## Abbreviations

LTR: Long Terminal Repeats
MITE: Miniature Inverted–repeat Transposable Elements
RT: Reverse Transcriptase
SINE: Short Interspersed Nuclear Elements
TE: Transposable element
