# Supplementary figures and images for: Mollusc genomes reveal variability in patterns of LTR-retrotransposons dynamics
Source: BMC Genomics. 2018 Nov 15;19:821. doi: 10.1186/s12864-018-5200-1 (PMC6238403; doi:10.1186/s12864-018-5200-1)

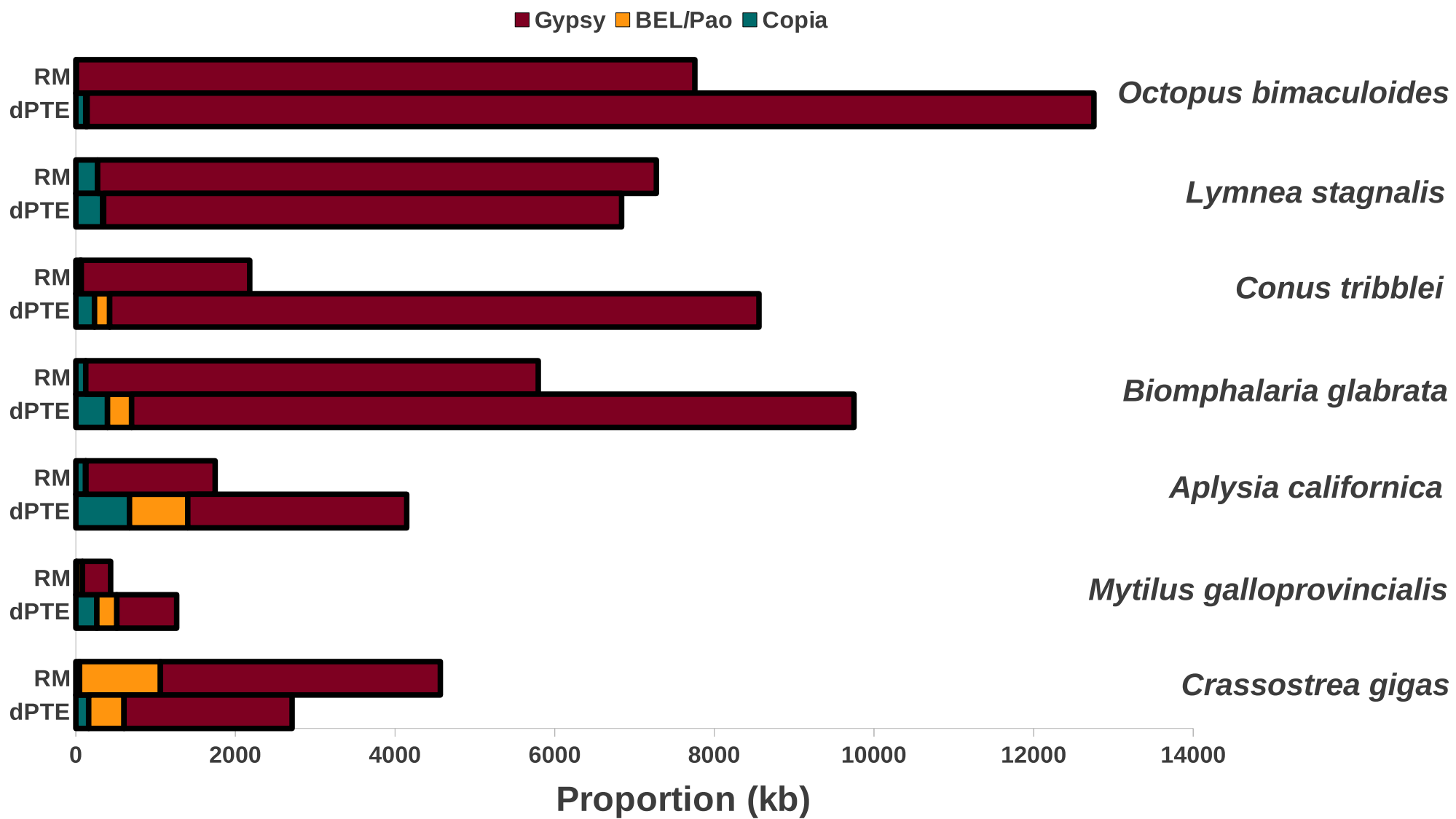

Supplement: Supplementary file 2 — Comparison of relative LTR-retrotransposon’s content estimated with RepeatMasker on assembled genome or dnaPipeTE using reads mapping. The horizontal axis indicates the abundance in kb of Copia (turquoise), BEL/Pao (orange) and Gypsy (maroon) superfamilies in each genome. (PDF 355 kb) [file 12864_2018_5200_MOESM2_ESM.pdf]
