# Supplementary material for: Mollusc genomes reveal variability in patterns of LTR-retrotransposons dynamics
Source: BMC Genomics. 2018 Nov 15;19:821. doi: 10.1186/s12864-018-5200-1 (PMC6238403; doi:10.1186/s12864-018-5200-1)
Supplement: Supplementary file 5 — Phylogenetic relationships among Gypsy clades. This tree is a simplified representation of Fig. 5, in which mollusc elements from a same clade are represented by compressed subtrees. All LTR-retrotransposon from a clade found in mollusc are depicted in color. The reference Gypsy elements and Gypsy clades previously reported in the Gypsy Database are in black. Node statistical support (> 65%) was obtained through non-parametric bootstrapping using 100 replicates. (PPTX 67 kb) [file 12864_2018_5200_MOESM5_ESM.pptx]

## Slide 1
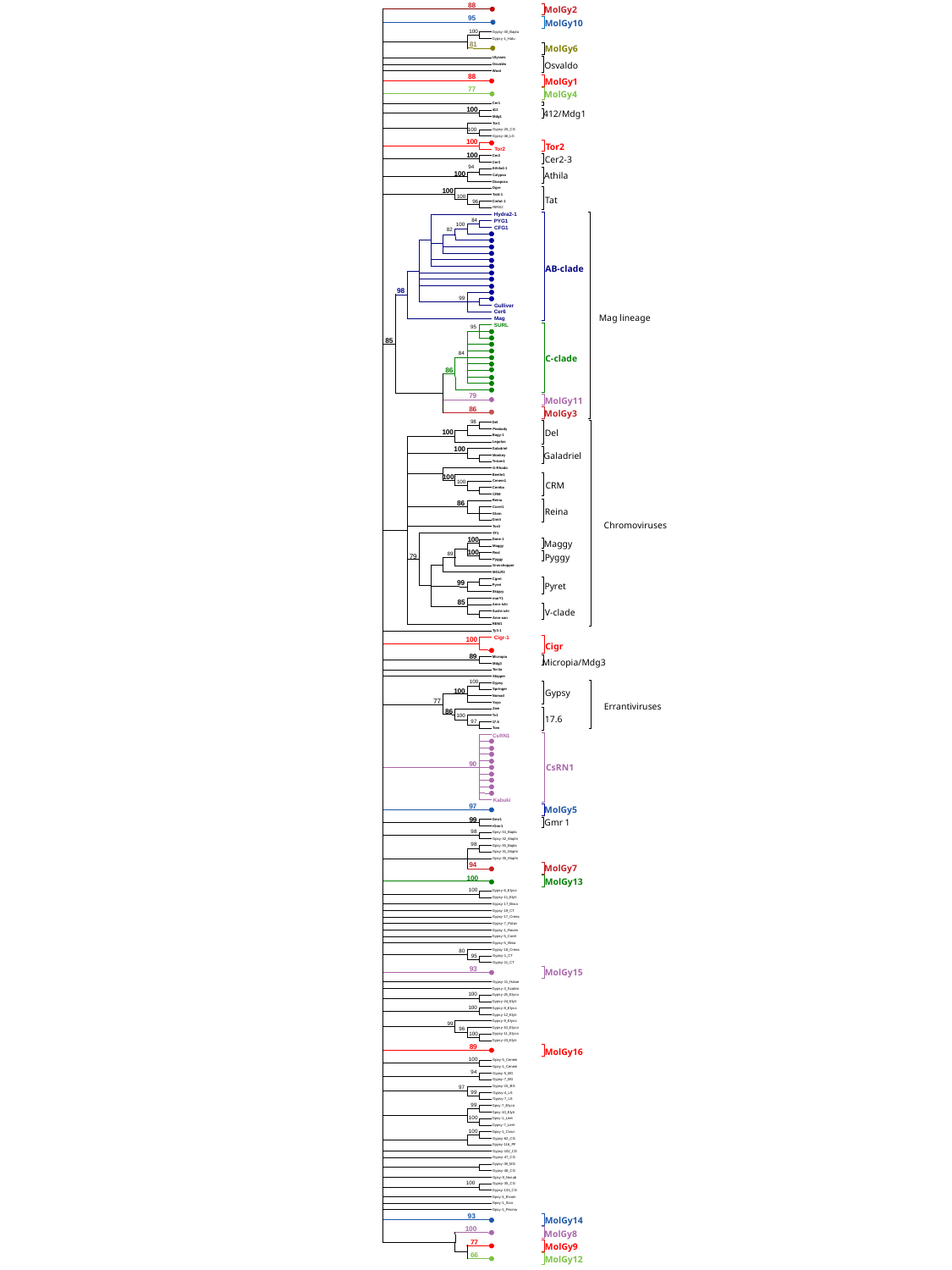

88
MolGy2
95
MolGy10
100
 Gypsy-30_Bapla
 Gypsy-1_Hatu
81
MolGy6
 Ulysses
Osvaldo
 Osvaldo
 Woot
88
MolGy1
77
MolGy4
 Cer1
100
 412
412/Mdg1
 Mdg1
 Tor1
100
 Gypsy-25_CG
 Gypsy-46_LG
100
Tor2
 Tor2
100
 Cer2
Cer2-3
 Cer3
94
 Athila4-1
100
Athila
 Calypso
 Diaspora
 Ogre
100
 Tat4-1
100
Tat
96
 Cinful-1
 RIRE2
 Hydra2-1
84
 PYG1
100
 CFG1
82
AB-clade
98
99
 Gulliver
 Cer6
Mag lineage
 Mag
 SURL
95
85
84
C-clade
86
79
MolGy11
86
MolGy3
98
 Del
 Peabody
100
Del
 Bagy-1
 Legolas
100
 Galadriel
Galadriel
 Monkey
 Tntom1
 G-Rhodo
 Beetle1
100
100
 Cenem1
CRM
 Cereba
 CRM
 Reina
86
 Coret1
Reina
 Gloin
 Gimli
Chromoviruses
 Tse3
 TF1
100
 Dane-1
Maggy
 Maggy
100
 Real
89
Pyggy
79
 Pyggy
 Grasshopper
 MGLR3
 Cgret
99
Pyret
 Pyret
 Skippy
 marY1
85
 Amn-ichi
V-clade
 Sushi-ichi
 Amn-san
 REM1
 Ty3-1
 Cigr-1
100
Cigr
89
 Micropia
Micropia/Mdg3
 Mdg3
 Tor4a
 Skipper
100
 Gypsy
100
 Springer
Gypsy
 Nomad
77
 Yoyo
Errantiviruses
 Zam
86
100
 Tv1
17.6
97
 17.6
 Tom
 CsRN1
90
CsRN1
 Kabuki
97
MolGy5
99
 Gmr1
Gmr 1
 rGmr1
98
 Gpsy-34_Bapla
 Gpsy-32_Mophi
98
 Gpsy-35_Bapla
 Gpsy-31_Mophi
 Gpsy-30_Mophi
94
MolGy7
100
MolGy13
100
 Gypsy-6_Elyco
 Gypsy-11_Elyti
 Gypsy-17_Bisia
 Gypsy-19_CT
 Gypsy-17_Crena
 Gypsy-7_Potan
 Gypsy-1_Raven
 Gypsy-5_Coret
 Gypsy-5_Bisia
 Gypsy-18_Crena
80
95
 Gypsy-1_CT
 Gypsy-11_CT
93
MolGy15
 Gypsy-11_Halae
 Gypsy-3_Scabro
100
 Gypsy-25_Elyco
 Gypsy-24_Elyti
100
 Gypsy-8_Elyco
 Gypsy-12_Elyti
 Gypsy-9_Elyco
99
 Gypsy-10_Elyco
96
100
 Gypsy-11_Elyco
 Gypsy-23_Elyti
89
MolGy16
100
 Gpsy-5_Cenem
 Gpsy-1_Cenem
94
 Gypsy-5_BG
 Gypsy-7_BG
97
 Gypsy-15_BG
99
 Gypsy-4_LS
 Gypsy-7_LS
99
 Gpsy-7_Elyco
 Gpsy-10_Elyti
100
 Gpsy-1_Liret
 Gypsy-7_Liret
100
 Gpsy-1_Cravi
 Gypsy-62_CG
 Gypsy-116_PF
 Gypsy-102_CG
 Gypsy-47_CG
 Gypsy-39_MG
 Gypsy-48_CG
 Gpsy-9_Nosub
100
 Gypsy-35_CG
 Gypsy-133_CG
 Gpsy-4_Elcom
 Gpsy-1_Sico
 Gpsy-1_Pecma
93
MolGy14
100
MolGy8
77
MolGy9
66
MolGy12
